# Melatonin as a Circadian Marker for *Plasmodium* Rhythms

**DOI:** 10.3390/ijms25147815

**Published:** 2024-07-17

**Authors:** Bárbara K. M. Dias, Abhinab Mohanty, Célia R. S. Garcia

**Affiliations:** Department of Clinical and Toxicological Analyses, School of Pharmaceutical Sciences, University of Sao Paulo, Sao Paulo 05508-000, SP, Brazil; bkmdias@gmail.com (B.K.M.D.); workabhinab@gmail.com (A.M.)

**Keywords:** *Plasmodium*, melatonin, circadian rhythm, PLC-IP3, calcium signaling

## Abstract

*Plasmodium*, a digenetic parasite, requires a host and a vector for its life cycle completion. Most *Plasmodium* species display circadian rhythmicity during their intraerythrocytic cycle within the host, aiding in immune evasion. This rhythmicity, however, diminishes in in vitro cultures, highlighting the importance of host-derived signals for synchronizing the parasite’s asexual cycle. Studies indicate a species–specific internal clock in *Plasmodium*, dependent on these host signals. Melatonin, a hormone the pineal gland produces under circadian regulation, impacts various physiological functions and is extensively reviewed as the primary circadian marker affecting parasite rhythms. Research suggests that melatonin facilitates synchronization through the PLC-IP_3_ signaling pathway, activating phospholipase C, which triggers intracellular calcium release and gene expression modulation. This evidence strongly supports the role of melatonin as a key circadian marker for parasite synchronization, presenting new possibilities for targeting the melatonin pathway when developing novel therapeutic approaches.

## 1. Introduction

Malaria continues to be a significant global health issue, affecting millions over the years. According to the 2023 WHO World Malaria Report, approximately 249 million cases were recorded, surpassing pre-pandemic estimates [1]. Malaria is caused by apicomplexan parasites of the genus *Plasmodium*. The four primary species that infect humans are *P. falciparum*, *P. vivax*, *P. malaria,* and *P. ovale*, with *P. knowlesi* also increasingly causing human infections due to zoonotic transmission from simian hosts [2,3].

The contemporary world grapples with the ongoing challenge of devising an effective treatment for this disease. Antimalarial resistance development by the parasite [4,5] and its vector, the *Anopheles* mosquito [6,7], coupled with the current antimalarial vaccines in use providing partial protection to the people [8,9,10], continues to maintain a high disease burden, especially among pregnant women and children [11]. The complexity of the parasite’s life cycle, including its sexual phase in mosquitoes and asexual phase in humans, complicates efforts to control malaria [12].

The life cycle of the *Plasmodium* parasite initiates when an infected *Anopheles* mosquito bites a human, injecting sporozoites that travel to the liver and invade hepatocytes. In the hepatocytes, the sporozoites multiply asexually, a phase known as exoerythrocytic schizogony [13]. After several days, hepatic schizonts rupture, releasing merozoites into the bloodstream. These merozoites infect red blood cells and alter their cytoskeletal structure to facilitate parasite growth [14], marking the start of the intraerythrocytic cycle, responsible for malaria’s clinical symptoms [15]. This cycle lasts 48 or 72 h in humans, depending on the *Plasmodium* species [16]. During this period, the parasite transforms through ring, trophozoite, and schizont stages [17]. Some merozoites develop into male and female gametocytes, which mature over 11 days [18,19].

Subsequently, the gametocytes must be acquired by the Anopheline mosquito during a blood meal from the infected metazoan host, marking the onset of the sporogonic cycle. In the mosquito’s digestive system, gametocytes differentiate into microgametes (male) and macrogametes (female). The ensuing fusion of these gametes gives rise to a diploid zygote which later evolves into a motile ookinete. The penetration of the ookinete into the mosquito’s midgut wall culminates in the growth of oocysts, which mature and rupture to liberate sporozoites. These sporozoites travel from the midgut and ultimately reside in the mosquito’s salivary glands, poised to be transmitted into its metazoan host during the next blood meal [17,18].

A remarkably distinctive occurrence unfolds during the intra-erythrocytic maturation of the parasite in vivo. As the asexual stages progress through the cycle, there is a pronounced tendency for parasites to simultaneously rupture from the host red blood cells (RBCs). This synchronized rupture occurs in time period multiples of 24 h depending on the parasite species [16], aligning with the host’s circadian rhythm [20]. The intriguing synchrony is theorized to confer several advantages to parasite survival by either eluding or overwhelming the immune system [21]. This coordinated schizont rupture is intricately linked to the paroxysm observed in a malaria patient [22]. Yet, the mechanisms that modulate synchronicity remain a focal point of current research.

## 2. *Plasmodium* Rhythms

The most visible fact demonstrating the need for a host marker to achieve parasite rhythm is that the in vitro cultivation of *Plasmodium* invariably leads to asynchronous parasite development [23] and requires intervention to achieve short-term synchronization [24], pointing to the fact that host factors are involved in the synchronization process. A seminal hypothesis presented by Hawking in 1970 suggested that *Plasmodium* parasites have developed rhythmic schizogony synchronized with the host circadian cycle, strategically enhancing parasite fitness and survival [25]. Evidence indicates that circadian adaptation facilitates biological needs by promoting coordination between individuals and their environment [26,27]. This holds particularly true for *P. falciparum*, where perturbation of the parasite rhythms to the host affects parasite vitality [28]. Also, the subsequential loss of synchrony increases the parasite’s vulnerability to drug treatments [29].

The Hawkings hypothesis was reinforced through subsequent experiments conducted by various research teams. Notably, one experiment revealed synchronicity between the peak infectivity window of a few *P. chabaudi* gametocytes circulating in mice and the mosquito feeding behavior [30]. Moreover, a recent study by Habtewold et al. showcased that the vectorial capacity of the anopheline mosquito varied diurnally with the highest susceptibility to infection in the evening, which correlated to the natural feeding time of the vector, highlighting its dependency on the circadian rhythm. It was observed that gametocytes exhibited inefficacy in forming oocysts within the mosquito midgut when subjected to reversed or abolished light–dark cycles. Additionally, when essential circadian genes such as *clock* were silenced, a similar decline in gametocyte infectivity and oocyst load was noted [31]. A similar pattern was observed in avian *Plasmodium* species, where reversing the light–dark cycles caused a reversal in the asexual cycle, leading to erythrocyte rupture during the daytime instead of the typical schizogony in the evening [32]. The avian model also established the conventional daily behavior to coincide with the peak period of transmission into its vector [33]. All evidence suggests a temporally tuned mechanism influenced by circadian signals, interconnected through the internal clocks of *Plasmodium*, its host, and its vector, which facilitates the proliferation of this digenetic parasite.

## 3. Do *Plasmodium* Parasites Possess an Intrinsic Clock?

Many researchers have argued that the parasite depends on its internal transcriptional regulation governing its rhythmicity. High-density time-series transcriptomic analysis in *P. falciparum* strains uncovered periodic rhythmicity in 87.3% to 92.5% of the mapped transcriptome in an in vitro system devoid of canonical host circadian signals. The parasites displayed conserved gene expression patterns akin to circadian systems, with strain-specific variations in period lengths suggesting genetic determination, challenging the notion of external influence on cycle duration [34]. The malaria parasite possessing an internal clock is further supported by another study where malaria parasite rhythms persisted despite constant darkness and in arrhythmic mice (4084 out of the 5244 genes under study). Conversely, nearly 20% of the studied transcriptome (999 out of the 5244 genes under study) ceased cycling within the arrhythmic hosts.

Further experimentation in arrhythmic mutant mice infected with *Plasmodium*, along with mathematical modeling, demonstrated a slow decay of synchrony, whereas the parasites infecting wild-type mice remained synchronous. These results suggest that the parasite rhythms are driven by an intrinsic clock, which depends on host cues to remain synchronized [35]. A similar trend was observed for the rodent strain, where 57% of *Plasmodium chabaudi* show a 24 h circadian periodicity during transcription. Disrupting parasite genes, particularly SR10, shortened the intraerythrocytic developmental cycle. The study could not disregard the impact of host signals, as parasites out of synchronization with the rodent’s circadian cycle also exhibited a loss of periodicity in 1765 of their genes, accounting for about 58% of the genes that showed 24-h rhythmic transcription [36].

In mammals, the circadian clock is regulated by a feedback loop between the transcription and translation of proteins from the core clock, whose products are essential for the maintenance of circadian rhythms in the cells, taking approximately 24 h to complete the feedback loop [37,38]. Besides the regulation of the intrinsic circadian clock by the transcription–translation loop at the cellular level, long-lasting regulation of circadian rhythms at tissue levels requires synchronizing cues, such as circadian circulation of hormones in the bloodstream or temperature cycles [39].

## 4. Host Cues Involved in *Plasmodium* Synchronization

There is ongoing discussion about how host cues, such as feeding patterns, influence parasites’ intra-erythrocytic cycle timing. These patterns are thought to synchronize the parasite’s asexual cycle with the host’s blood glucose fluctuations. Parasites adjust to changes in host feeding schedules, suggesting that they have mechanisms to control this synchronization [40]. Glucose levels in the host exhibit a daily cycle, regulated by insulin in hepatocytes, and result in patterns of nocturnal hypoglycemia and morning hyperglycemia, which occur independently of external cues [41].

The parasite replication has been demonstrated to be linked to the periodic release of nutrients during digestion, which is influenced by host feeding patterns. Notably, this association persists irrespective of signals from core clock proteins such as PERIOD 1 and 2 [42]. *P. chabaudi* also exhibits periodic plasticity in the intraerythrocytic developmental cycle in response to a 12 h mismatch in host feeding–fasting rhythms. The parasite advances each asexual cycle by 2–3 h without experiencing significant fitness loss, enabling it to catch up to the host rhythms [43]. Interestingly, aligning with feeding–fasting rhythms appears advantageous for parasites in hosts with functional circadian clocks. However, parasites do align in hosts with perturbed circadian cues without availing any benefit [44]. Moreover, parasite rhythms are subdued in clock-mutant hosts that continuously consume food [42]. This evidence suggests that food cues may serve as indirect markers rather than direct selective drivers of parasite rhythm. Despite that, the interplay between the host immune response, glucose metabolism, and nutrient availability regulates schizogony timing in *Plasmodium* infection [45]. During *P. chabaudi* infection, IFNγ-primed leukocytes exhibit enhanced glucose metabolism and pro-inflammatory gene expression, while TNFα-induced hypoglycemia restricts parasite replication. Disruptions in cyclic hypoglycemia and *P. chabaudi* stage synchronization occur in IFNγ-deficient, TNFα-deficient, or diabetic mice [46].

Studies investigating rhythms in proteins present in the serum, plasma, and tissues altered in malaria and immune factors related to the disease analyzed published data sets from the literature and observed that many immune factors and host response pathways are under circadian control. Data from *P. falciparum* and *P. vivax* infections in humans indicate 73 overlapping rhythmic proteins from circadian regulated pathways under healthy conditions, such as complement and coagulation cascade. In addition, the analysis showed that rhythmic immune factors present in humans, mice, and baboons infected with malaria parasites participate in essential host immune response pathways, such as leukocyte activation and cytokine production. These data indicate that the host circadian rhythms coordinate immunological networks and host responses in malaria infection [47].

Using a mouse model of cerebral malaria, Cabral et al. (2024) investigated the effects of host circadian regulation in mice infected with *P. berghei* ANKA. The authors evaluated the host response and parasite growth when infection was performed at different times of the day and showed that the time of infection directly affects the parasite growth, which might be a consequence of the daily variation in the number of reticulocytes (parasite-targeted cells) in the host. In addition, the authors observed that the parasite load presents a 24 h rhythm that can be affected by different environmental and host cues, such as prolonged jetlag, sucrose administration, and feeding rhythms [48].

A recent clinical investigation indicates that the alignment of the asexual phase of the parasite with the host’s circadian phases is not consistent or immediate. Instead, it seems to be a dynamic and time-sensitive occurrence that differs among individuals and throughout various infection stages [49].

## 5. Melatonin Circadian Production and Release by the Pineal Gland

Melatonin, or N-acetyl 5-metoxytryptamine, is ubiquitous in bacteria, fungi, animals, and plants [50]. Due to its structure, melatonin is a potent free radical scavenger and contributes to protection against oxidative stress. Its chemical structure allows the hormone to interact with oxygen and nitrogen-reactive species and thus melatonin’s antioxidant function is present in all organisms in which the hormone is present [51]. The presence of melatonin in primitive organisms points to the fact that the primary function of this hormone would be the antioxidant activity contributing to combatting the free radical formation resultant from aerobic metabolism [52]. Interestingly, melatonin metabolism, once it interacts with oxygen and nitrogen reactive species results, in the generation of metabolites that also function as free radical scavengers, resulting in a free radical scavenging cascade making melatonin a potent antioxidant [52,53].

Besides melatonin’s function as an antioxidant, it plays a vital role in regulating different cellular and physiological processes [45]. In humans, this hormone is one of the significant markers of the endogenous circadian clock; its production by the pineal gland is mainly controlled by the light–dark cycle and alterations in the daylight and darkness [54]. Melatonin synthesis by the pineal gland directly reflects its levels in the blood since no melatonin is stocked in the pinealocytes due to its lipophilic feature [55].

The canonical pathway for melatonin production in the pineal gland starts with the photoreceptors in the retina, which receive information about the light–dark cycle and trigger a conformational change in melanopsin, the photopigment present in retinal ganglion cells. This information is then transduced in nervous signals through the SCN to the pineal gland, where the melatonin synthesis is performed [56,57]. Due to the light–dark regulation of melatonin production, the levels of this hormone in the bloodstream start to increase in the beginning of the evening and reach their peak in the middle of the night [54,58].

Melatonin synthesis by pinealocytes occurs through the essential amino acid tryptophan, obtained from the diet, which is present in the blood and is absorbed by the pinealocytes, where the amino acid is converted into melatonin in four steps. The first step is the hydroxylation of tryptophan in carbon 5 of the indole ring by the enzyme tryptophan hydroxylase. Next, the 5-hydroxytryptophan is decarboxylated by the 5-hydroxytryptophan decarboxylase, deriving the serotonin. Serotonin is then converted to N-acetyl-serotonin in the limiting step of the pathway by the arylalkylamie N-acetyltransferase (AANAT). This limiting aspect of melatonin synthesis by AANAT is given by the modulation of AANAT expression by the light. In the presence of light, AANAT expression is decreased, inhibiting melatonin production [59]. Next in the melatonin synthesis, N-acetyl-serotonin is converted to the final product, melatonin, by the N-acetylserotonin-O-methyl transferase, adding a methoxy group in the carbon five of the N-acetylserotonin indole ring.

Melatonin production in pinealocytes is modulated by circadian timing and light and dark cycles. Thus, melatonin is one of the major markers of circadian rhythms and plays an important role in maintaining the endogenous circadian clock in humans [58].

## 6. Melatonin as a Circadian Host Cue to Synchronize *Plasmodium* Rhythms

Melatonin levels in the blood are circadian-coordinated and vary according to the light/dark cycle, thus being a significant marker of circadian rhythms. Hotta et al. (2000) [60] investigated the role of this host hormone in the erythrocytic development of *Plasmodium*. The authors compared the intraerythrocytic development of the rodent parasite *Plasmodium chabaudi* in mice pinealectomized or not and observed that parasites infecting pinealectomized mice could not develop synchronously. In contrast, in non-pinealectomized mice, the proportion of parasites in the trophozoite stage was significantly higher when compared to schizont or rings, implying a synchronous development, which was also observed when pinealectomized mice were treated with exogenous melatonin. Moreover, the authors also showed that in an in vitro culture of the human parasite *Plasmodium falciparum*, 24 h treatment with 10–100 nM of melatonin resulted in a decrease in the percentage of parasites in the early stages of the intraerythrocytic cycle (ring and trophozoite) and an increase in the number of schizonts, pointing to the fact that melatonin treatment leads to parasite synchronization both in vitro and in vivo [60].

In the same study, Hotta et al. (2000) [60] showed that the addition of melatonin to erythrocytes infected with *P. chabaudi* loaded with the Ca^2+^ marker Fluo-4AM resulted in an increase in cytosolic Ca^2+^ concentration. To investigate whether this Ca^2+^ rise would result from intracellular Ca^2+^ mobilization, the authors used the chelator EGTA. They observed that in the absence of extracellular Ca^2+^, melatonin addition still triggers an increase in cytosolic Ca^2+^ (Ca^2+^[cyt]). This Ca^2+^ response to melatonin was abolished when cells were pretreated with the melatonin receptor inhibitor luzindole (LZD) or the phospholipase C (PLC) inhibitor U73122, showing that melatonin mobilizes Ca^2+^ from intracellular stocks by the activation of PLC, the enzyme responsible for cleaving phosphatidylinositol 4,5-bisphosphate (PIP2) into inositol triphosphate (IP_3_) and diacylglycerol (DAG) upon activation of the GPCR involved in the IP_3_-DAG signaling pathway [60].

Bagnaresi et al. (2009) [61] confirmed the role of host-derived melatonin in *Plasmodium* rhythmicity in asexual development. The authors demonstrated that *Plasmodium* species that remain asynchronous in their hosts, like *P. berghei* and *P. yoelii*, do not respond to the addition of melatonin, evidenced by the absence of downstream signal activation like the release of intracellular calcium or modulation of the cell cycle towards a synchronous growth [61].

Beraldo et al. (2007) [62] used the IP_3_ modulator and inhibitor of store-operated Ca^2+^ channels (SOCs), 2-Aminoethyl diphenylborinate (2-APB), to investigate the mechanism of melatonin signaling in *P. falciparum*. The authors reported that in parasites treated with 2-APB along with melatonin, the increase in the number of schizonts is abolished when compared to parasites treated with melatonin alone. The study also showed that the addition of 2-APB to Fluo4-AM-labeled parasites in the trophozoite stage does not result in a Ca^2+^[cyt] increase but prevents the Ca^2+^ rise in response to melatonin addition, as well as treatment with LZD and the PLC inhibitor, indicating that IP_3_ production and IP_3_-modulated calcium stocks are involved in the melatonin signaling pathway in *Plasmodium* [62].

Using cell-permeant caged-IP_3_, Alves et al. (2011) investigated the IP3-dependent release of Ca^2+^ in *P. falciparum*-infected erythrocytes. The release of IP_3_ by UV flashes resulted in a transient increase in Ca^2+^[cyt] concentrations. In addition, the authors reported that *P. falciparum* trophozoite stage parasites stimulated with melatonin elicit Ca^2+^ from internal stores, and for most of the observed cells, sequential Ca^2+^ release after photo release of caged IP_3_ was not observed, suggesting that melatonin also mobilizes Ca^2+^ from the ER [63].

The melatonin signaling cascade in *Plasmodium falciparum* involves intricate interactions between the second messengers Ca^2+^ and cAMP. During the trophozoite stage, Beraldo et al. (2005) found that melatonin treatment increases cAMP levels in *P. falciparum*. Still, this increase can be blocked by the PLC inhibitor U73122 and the intracellular Ca^2+^ chelator BAPTA, suggesting that Ca^2+^ levels influence cAMP production in response to melatonin. Furthermore, treatment with melatonin and protein kinase inhibitor peptide (PKI), which blocks the activity of the protein kinase A (PKA), reduced the increase in schizont percentage typically seen with melatonin treatment. The study also revealed that when exposed to melatonin, isolated trophozoites exhibit increased PKA activity. Notably, the rise in cytosolic Ca^2+^ induced by melatonin precedes PKA activation, as demonstrated by the fact that PKI treatment did not prevent the increase in Ca^2+^[cyt]. Additionally, the study highlighted the interaction between the two second messengers, indicating that cAMP can prompt Ca^2+^ mobilization from the same intracellular stores as melatonin, leading to a PKA-dependent increase in Ca^2+^[cyt] [64].

Interestingly, Furuyama et al. (2014) showed that treatment with the melatonin receptor antagonist luzindole completely abolished spontaneous oscillations of Ca^2+^ in parasites in the ring stage, but not in early trophozoites. In addition, in vitro treatment with 250 µM luzindole delayed the intraerythrocytic development of *P. falciparum* FCR-3. Moreover, treatment with the adenylyl cyclase inhibitor N-(Cis-2-phenyl-cyclopentyl) azacyclotridecan-2-imine-hydrochloride (MDL 12330A) also impaired parasite development in vitro [65].

However, studies have already identified some effector proteins that play an essential role in the melatonin effect in the parasite erythrocytic development. Koyama et al. (2012) reported the involvement of the *P. falciparum* atypical kinase PK7 in the melatonin signaling pathway in the parasite. Using a parasite strain knockout for PfPK7 (PfPK7^−^), the authors observed that treatment with different concentrations of melatonin (0.1, 1, and 10 µM) in PfPK7^−^ does not increase the percentage of parasites in the schizont stage, as observed in wild-type 3D7 parasites. However, this effect could be partially recovered by the PfPK7 complement. Furthermore, the knockout of *pfpk7* resulted in a diminished Ca^2+^ [cyt] increase when compared to wild-type parasites, pointing out that this kinase possesses an essential role in the melatonin effect in the erythrocytic development of *P. falciparum* [66].

In *Plasmodium falciparum*, the Eukaryotic Initiation Factor 2 Kinase 1 (PfeIK1) plays a pivotal role in the melatonin signaling pathway, primarily regulating protein translation during amino acid scarcity. Dias et al. (2020) showed that melatonin treatment affects the expression of the *pfeik1* gene, explicitly reducing its expression during the ring stages of the erythrocytic cycle in wild-type 3D7 parasites. Furthermore, their study noted increased parasitemia in these wild-type parasites treated with 100 nM melatonin for 48 h. Contrastingly, parasites deficient in PfeIK1 showed no such increase, highlighting the essential role of the PfeIK1 kinase in the melatonin signaling pathway of *P. falciparum*. The absence of this parasite kinase suggests that without PfeIK1, melatonin cannot effectively modulate parasite growth [67].

The Microrchidia (MORC) protein of *Plasmodium falciparum* is crucial in regulating the effects of melatonin on the parasite’s development. Research by Singh et al. (2021) revealed that melatonin alters MORC expression in ring-stage parasites, with notable gene expression changes within two hours of treatment. The study also examined the consequences of MORC knockdown on melatonin’s influence on the parasite’s erythrocytic stages, finding that melatonin’s impact was diminished in parasites with reduced MORC levels, especially in the development of schizonts in both wild-type and MORC knockdown 3D7 strains. These findings emphasize PfMORC’s significant role in melatonin pathways and its essential functions in chromatin structure organization and gene transcription, linked to interactions with APETALA2 (AP2) family transcription factors [68,69].

**Figure 1:**
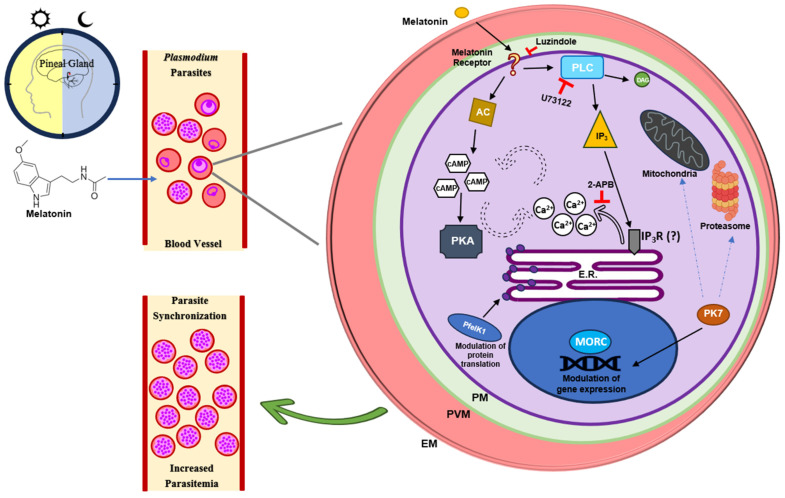
Host-secreted melatonin effect on *Plasmodium* parasites. Melatonin is secreted in a circadian manner by the pineal gland. The melatonin released into the bloodstream interacts with the *Plasmodium* parasites in infected erythrocytes, triggering a signaling cascade. Melatonin binds to a still unidentified melatonin receptor in *Plasmodium*, activating PLC and resulting in the production of IP_3_ and DAG. IP_3_ then activates a yet unknown IP_3_ receptor (IP_3_R), resulting in the release of Ca^2+^ from the endoplasmic reticulum to the cytosol. Concurrently, melatonin enhances cAMP production by adenylyl cyclase, leading to PfPKA activation. Additionally, the kinase PfPK7 plays a role in the melatonin pathway, modulating the expression of genes involved in the ubiquitin–proteasome system and mitochondria division. The nuclear protein MORC is also associated with melatonin-regulated gene expression and chromatin structure organization. Similarly, PfeIK1 involved in the regulation of protein translation plays a central role in the melatonin signaling pathway in *Plasmodium*. All the signaling pathways activated by melatonin in the parasite lead to increased parasitemia and synchronization of the asexual blood stages inside the host. PLC—Phospholipase C; IP_3_—Inositol-1,4,5-triphosphate; DAG—Diacylglycerol; AC—Adenylyl cyclase; ER—Endoplasmic reticulum; 2-APB—2-Aminoethoxydiphenyl borate; PM—Parasite membrane; PVM—Parasitophorous vacuolar membrane; EM- Erythrocyte membrane.

Furthermore, studies suggest that melatonin influences the expression of different genes in *P. falciparum*, initiating a signaling cascade that starts with the activation of PLC, followed by the release and production of the secondary messengers Ca^2+^ and cAMP, which, in turn, activate effector proteins and trigger gene transcription (Figure 1).

Lima et al. (2016) used transcriptomic analysis to show that 100 nM melatonin treatment for 5 h in trophozoite stage 3D7 parasites resulted in 38 differently expressed genes, with upregulated genes including the eucaryotic initiation factor 2, essential for protein translation, and also genes involved in RNA metabolism, zinc finger proteins, and genes coding for other proteins involved in protein translation [70]. Scarpelli et al. (2018) reported that melatonin treatment modulates mitochondria fission by modulating the expression of genes involved in the fission and division of mitochondria [71].

Interestingly, Kobayashi et al. (2023) identified an apicoplast RNA polymerase σ subunit, *apiSig*, with rhythmic expression, presenting a 48 h periodicity in *P. falciparum*. Due to the periodicity in *apSig* expression, the authors investigated the coordination of the gene expression with the host melatonin levels. Using RT-PCR, the authors showed that 10 nM melatonin treatment for 30 min or 90 min increases the transcript levels of *apSig* and modulates the expression of the apicoplast genes *rpoC2, sufB*, and *tufA*. The authors also showed that the effect of melatonin in the *apSig* expression is absent in ring stage parasites and is impaired by the adenylyl cyclase inhibitor MDL12330, implying that melatonin modulation of *apSig* could be stage-specific and dependent on cAMP production [72].

Extensive studies have revealed that apicomplexans like *Plasmodium* synthesize phosphoinositides (PIs), particularly Myo-inositol 1,4,5-trisphosphate (InsP3), although the InsP3 receptor remains unidentified in their genomes [73,74,75,76,77]. This review also suggests that melatonin initiates a complex signaling cascade in *Plasmodium falciparum*, potentially involving an unidentified melatonin receptor. This cascade triggers calcium release from the endoplasmic reticulum and alters gene expression, synchronizing the parasite’s development. This insight opens up the possibility of disrupting the synchronized growth of *Plasmodium* by identifying and targeting elements of this signaling pathway, offering a ray of hope in the fight against malaria.

**Figure 2:**
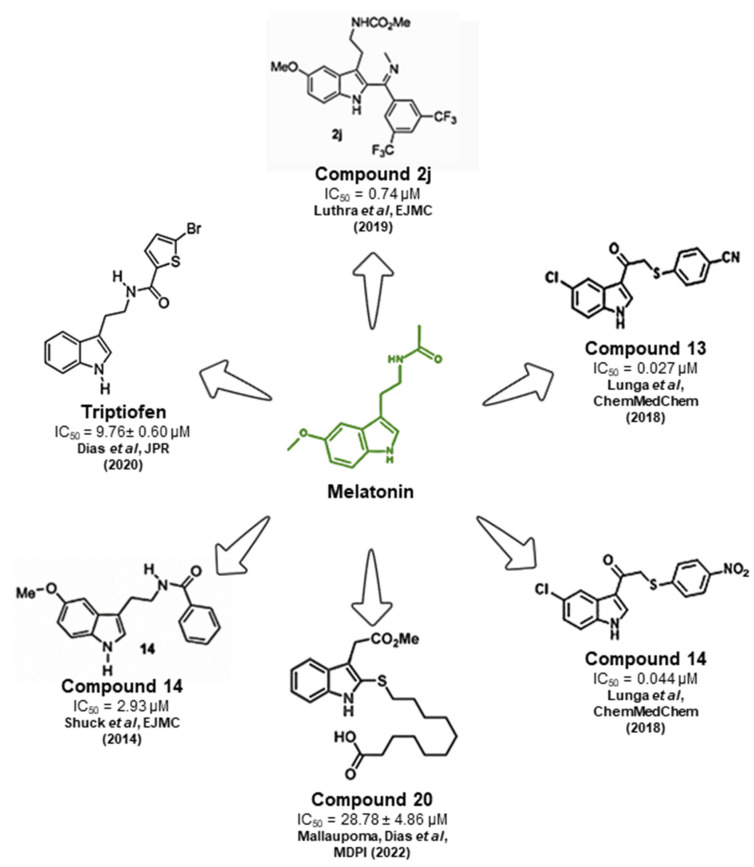
Indole compounds with promising activity against the asexual stages of malaria parasites. The schematic represents the melatonin structure (green) and melatonin-related indole compounds with promising antimalarial activity (black) (Compound 2j [80], Triptiofen [67], Compound 14 [78], Compound 20 [81], Compound 14 [79], & Compound 13 [79]) along with their respective structures and IC_50_ values from previously published work.

The research on melatonin receptor antagonists demonstrates their potential to impair the development of parasites within red blood cells, suggesting a promising avenue for new antimalarial drugs. Various studies have focused on compounds related to melatonin that target its pathway in the malaria parasite, aiming to disrupt the progression of the *Plasmodium* cycle (Figure 2).

Schuck et al. (2014) evaluated synthetic indole compounds, similar to melatonin, against the intraerythrocytic development of *P. falciparum* in vitro. These compounds, displaying IC_50_ values in the micromolar range and as low as 2.93 µM, highlighted the significance of the indole ring in new antimalarial developments [78].

Lunga et al. (2018) explored the indole scaffold to perform a structure–activity relation study, with substitutions in different regions of the indole ring. The authors observed that halogen substituents in the C5 of the indole ring, in addition to the methyl and methoxy group in this position, increased the activity against *P. falciparum* in vitro while keeping low cytotoxicity against HeLa cells [79].

Luthra et al. (2019) synthesized and assessed the activity of melatonin-based indole compounds against malaria, identifying compounds with low IC_50_ values ranging from 0.73 to 4.28 µM. These compounds were effective against chloroquine-resistant parasites and were non-toxic to HepG2 cells. Their most notable compound, 2j (0.74 µM IC_50_), also blocked melatonin-induced parasitemia and synchronization and inhibited melatonin’s binding to the human melatonin receptor MT1 [80].

Dias et al. (2020) explored synthetic indole compounds in *P. falciparum* and discovered a partial agonist to the melatonin pathway in the parasite named Melatotosil. This compound could increase parasitemia at nanomolar concentrations and block the effects of melatonin when co-administered with the hormone. This study underscored the critical role of the methoxy group at C5 of the indole ring in enhancing parasitemia, noting that a structurally similar compound without this methoxy group did not increase parasitemia when administered alone. These compounds also blocked parasite development in vitro in the micromolar range without being cytotoxic to HEK293 cells, with the lowest IC_50_ value being 9.76 µM [67].

More recently, Mallaupoma et al. (2022) studied 2-sulfenylindoles against the *P. falciparum* strains 3D7 and Dd2, resistant to chloroquine, in vitro. This research showed that these compounds had antimalarial activity in the micromolar range, with one particular compound (compound **20**) showing increased activity against the chloroquine-resistant strain Dd2. Notably, eight of the fourteen compounds tested exhibited a synergistic effect in increasing parasitemia when combined with melatonin [81].

These compelling findings underscore the potential of targeting the melatonin signaling pathway and ignite a sense of hope in developing new antimalarial compounds that are effective against resistant strains and have low toxicity to mammalian cells.

## 7. Conclusions

The synchronous development of *Plasmodium* parasites ensures infection progression in the host due to immune system evasion. The molecular players involved in the synchronization process are not entirely elucidated. Studies point out that the parasite possesses an intrinsic clock that maintains the synchronous development and synchronous transcription of genes. However, no *clock*-like genes have been identified in the parasite genome, thus, further efforts are necessary to elucidate the molecular mechanism behind the observed rhythmic behavior of the parasite. Moreover, parasites infecting arrhythmic hosts lose synchronicity over time, implying that this intrinsic clock is entrained by a host cue.

Many efforts have been made to identify the host cues involved in synchronization, and melatonin, a host hormone produced in a circadian way by the pineal gland, can trigger a complex signaling cascade, leading to an increase in the mature stages of the intraerythrocytic cycle and synchronization in vitro. Nevertheless, some gaps still need to be filled regarding the melatonin signaling pathway in *Plasmodium*. The upstream activation of the signaling pathway is still unknown as the melatonin receptor is yet to be identified in the parasite.

Impairing parasite rhythms can jeopardize the infection progression, thus making the parasite more susceptible to antimalarial compounds. This review provides ample evidence in elucidating the significance of the melatonin pathway in Plasmodium. Understanding this pathway can open up new avenues for fighting the rapid development of parasite resistance to the drugs currently used for disease treatment.
